# A Confluence of Policy Inequality: Health Disparities in Old Age Among American Indian and Alaska Native Populations

**DOI:** 10.1093/geroni/igaa057.120

**Published:** 2020-12-16

**Authors:** Sadie Giles

**Affiliations:** Virginia Tech, Blacksburg, Virginia, United States

## Abstract

Racial health disparities in old age are well established, and new conceptualizations and methodologies continue to advance our understanding of health inequality across the life course. One group that is overlooked in many of these analyses, however, is the aging American Indian/Native Alaskan (AI/NA) population. While scholars have attended to the unique health inequities faced by the AI/NA population as a whole due to its discordant political history with the US government, little attention has been paid to unique patterns of disparity that might exist in old age. I propose to draw critical gerontology into the conversation in order to establish a framework through which we can uncover barriers to health, both from the political context of the AI/NA people as well as the political history of old age policy in the United States. Health disparities in old age are often described through a cumulative (dis)advantage framework that offers the benefit of appreciating that different groups enter old age with different resources and health statuses as a result of cumulative inequalities across the life course. Adding a framework of age relations, appreciating age as a system of inequality where people also gain or lose access to resources and status upon entering old age offers a path for understanding the intersection of race and old age. This paper will show how policy history for this group in particular as well as old age policy in the United States all create a unique and unequal circumstance for the aging AI/NA population.

